# Increasing trends in primary NNRTI resistance among newly HIV-1-diagnosed individuals in Buenos Aires, Argentina

**DOI:** 10.7448/IAS.16.1.18519

**Published:** 2013-10-03

**Authors:** Nahuel Rodriguez-Rodrigues, Adriana Duran, María Belen Bouzas, Ines Zapiola, Marcelo Vila, Debbie Indyk, Emiliano Bissio, Horacio Salomon, Dario A Dilernia

**Affiliations:** 1 Instituto de Investigaciones Biomédicas en Retrovirus y SIDA (INBIRS), Universidad de Buenos Aires-CONICET, Buenos Aires, Argentina; 2 Coordinacion SIDA, Ministerio de Salud del Gobierno de la Ciudad Autonoma de Buenos Aires, Argentina; 3 Division Analisis Clinicos, Hospital “Dr. Francisco Javier Muñiz”, Argentina; 4 Unidad de Virologia, Hospital “Dr. Francisco Javier Muñiz”, Argentina; 5 PAHO/WHO Argentina; 6 Department of Preventive Medicine, Icahn School of Medicine at Mount Sinai, New York, US; 7 Dirección de Sida y ETS, National Ministry of Health, Argentina

**Keywords:** HIV, primary drug resistance, trend, NNRTI, newly diagnosed

## Abstract

**Objective:**

Our objective was to estimate primary resistance in an urban setting in a developing country characterized by high antiretroviral (ARV) coverage over the diagnosed population and also by an important proportion of undiagnosed individuals, in order to determine whether any change in primary resistance occurred in the past five years.

**Design:**

We carried out a multi-site resistance surveillance study according to WHO HIV resistance guidelines, using a weighted sampling technique based on annual HIV case reports per site.

**Methods:**

Blood samples were collected from 197 drug-naive HIV-1-infected individuals diagnosed between March 2010 and August 2011 at 20 HIV voluntary counselling and testing centres in Buenos Aires. Clinical records of enrolled patients at the time of diagnosis were compiled. Viral load and CD4 counts were performed on all samples. The *pol* gene was sequenced and the resistance profile determined. Phylogenetic analysis was performed by neighbour-joining (NJ) trees and bootscanning analysis.

**Results:**

We found that 12 (7.9%) of the 152 successfully sequenced samples harboured primary resistance mutations, of which K103N and G190A were the most prevalent. Non-nucleoside reverse transcriptase inhibitors (NNRTI) resistance mutations were largely the most prevalent (5.9%), accounting for 75% of all primary resistance and exhibiting a significant increase (*p*=0.0072) in prevalence during the past 10 years as compared to our previous study performed in 1997–2000 and in 2003–2005. Nucleoside reverse transcriptase inhibitor (NRTI) and protease inhibitor primary resistance were low and similar to the one previously reported.

**Conclusions:**

Levels of primary NNRTI resistance in Buenos Aires appear to be increasing in the context of a sustained ARV coverage and a high proportion of undiagnosed HIV-positive individuals.

## Introduction

Highly active antiretroviral therapy (HAART) can increase life expectancy of HIV-positive patients [1–4] and prevent transmission [5]. However, it may also drive the selection of mutations that confer resistance to antiretroviral (ARV) drugs in patients under treatment. Transmission of drug-resistant variants constitutes a public health issue since it can contribute to failure of first-line ARV treatment [6]. Prevalence of primary resistance appears to be correlated with treatment coverage, being higher in developed settings [7–19]. In fact, international guidelines recommend performing genotypic resistance testing in all drug-naïve patients, before beginning a first-line ARV regimen [20–22].

Although by the end of the 1990s, improvements in ARV regimens had limited the rate of selection and transmission of resistance mutations (mainly for those associated with nucleoside reverse transcriptase inhibitor (NRTI) resistance), significant increasing trends in primary resistance have been reported in the past decade in settings where HAART regimens are broadly implemented: increasing trends appear to be observed exclusively in developed settings, such as the United Kingdom [23], Germany [24] and the United States [25], but not in developing settings [26–28]. This might be associated with the fact that the high proportion of undiagnosed individuals in developing countries may limit population-level selective forces driven by ARV treatment. It is not clear whether the low prevalence of primary resistance in these settings will remain stable or will increase at a slower rate than that of the developed settings.

Argentina is a developing country with approximately 130,000 individuals living with HIV, with half of them unaware of their infection [29]. Free access to ARV drugs has been guaranteed since 1992, and Argentina, together with Brazil, was the first Latin American countries that established an ART delivery programme. Buenos Aires, the capital city, is the most populated, containing 47.1% of reported HIV cases between 2001 and 2010 [30]. In Buenos Aires, there are 11,124 people receiving ARV treatment with 67% of them taking NRTI drugs: 40% (4442) receive AZT/3TC, 13% (1434) ABC/3TC, 11% (1250) TDF/FTC and 3% (305) TDF/3TC. A 52% (5738) receive NNTRI drugs, 40% (4435) efavirenz and 12% (1303) with nevirapine, and 38% (4232) take at least one protease inhibitor, of which 454 are not reinforced with ritonavir.

In the setting of Buenos Aires city, we have previously reported a prevalence of primary resistance among newly diagnosed individuals of 2.3% (CI: 0–5.5, *n*=86) in 1997–2000 [31] and 4.2% (CI: 1.9–6.5, *n*=284) in 2003–2005 [32]. In addition, a prevalence of 7.7% (CI: 0.4–14.9, *n*=52) was reported for individuals recently infected in 2004–2005 [33]. Importantly, non-nucleoside reverse transcriptase inhibitors (NNRTI) resistance mutations (mainly K103N) were not found in the first study but were detected in the last two studies.

In the present report, we study individuals recently diagnosed between 2010 and 2011 and show that the increasing trend in overall primary resistance persists driven mainly by the accumulation of NNRTI-associated resistance mutations.

## Materials and methods

### Study design

This resistance surveillance study was designed according to WHO HIV resistance guidelines [34]. These guidelines do not make restrictions in age as the 2008 updated guidelines do. This allows us to compare our results with our previous studies and also to reach a higher sample size. A comparison with results obtained following the 2008 updated guidelines is provided in this article. In brief, using a cross-sectional weighted sampling technique, newly HIV-diagnosed individuals were sequentially selected at the time of diagnosis at 20 health centres that include voluntary and counselling testing sites (VCTs) and infectious diseases services from public hospitals and private clinics. The proportion of individuals included from each site reflected the proportion of new cases reported to the Ministry of Health from 2003 to 2009.

### Study population

The inclusion criteria were being HIV positive, age older than 18, with first confirmed diagnosis of HIV infection within a period of 180 days before study entry, with confirmed unexposure to ARV therapy and agreement to participate in the study by signing an informed consent. A total of 197 drug-naïve newly diagnosed HIV-1 individuals were enrolled between March 2010 and August 2011, and those who met the inclusion criteria were included in the study. The final study population (N=152) comprised 116 men, 33 women and 3 transsexual, of whom 28 (18%) were younger than 25 years, with an overall average age of 37 years.

### Ethical approval and details of the consent procedure for the study

The project was initially submitted to the Bioethics Committee of the Muñiz Hospital specialized in infectious diseases in Buenos Aires, Argentina. It is a UNESCO-recognized committee and is listed among the ethics institutions list available at http://www.unesco.org/new/en/social-and-human-sciences/themes/global-ethics-observatory/access-geobs/. The Bioethics Committee of the Muñiz Hospital approved the project on 16 November 2009. The project was later validated by the Academic and Scientific Direction of the Government of the City of Buenos Aires on 29 September 2010. Patients were asked to read and, if agree with participating in the study, to sign an informed consent form. In this form, the patient can read in an accessible language the objectives of the project and the assays to be performed over the collected samples.

### HIV-1 RNA isolation and sequencing

RNA was isolated from plasma by the QIAamp viral extraction Kit (QIAGEN GmbH, Hilden, Germany). The *pol* gene was amplified between positions 2142 and 3798 (reference strain HXB2 numbering [35]) by reverse transcriptase-polymerase chain reaction (RT-PCR) and was sequenced by ABI Prism 3100/3100-Avant equipment (Applied Biosystems, Foster City, CA, USA).

For *pol* gene amplification, outer primers 5CP1 (5′-GAAGGGCACACAGCCAGAAATTGCAGGG-3′) and RT3.1 (5′-GCTCCTACTATGGGTTCTTTCTCTAACTGG-3′), and inner primers 1F (5′-CAGACCAGAGCCAACAGCCCC-3′), A35 (5′-ATTGGTTGCACTTTAAATTTTCCCATTAGCCCTATT-3′), 6B (5′-CATTGTTTAACTTTTGGGCC-3′), RT3208F (5′-AACATCAGAAAGAACCTCCATT-3′), NE1 (5′-CGACCTGACAGTTACTGTATGTCTTCAATCACC-3′) and 6B (5′-CATTGTTTAACTTTTGGGCCATCCATTCCTGGC-3′) were used.

The reverse transcription reaction was performed by heating at 42°C for 50 minutes and 70°C for 15 minutes using the Superscript II RT enzyme and the RT3.1 primer. *Pol* gene amplification was performed by nested PCR using the primers listed above. The PCR conditions were 3 first minutes at 95°C, then 5 cycles of 15 seconds of denaturation at 95°C, 15 seconds of primer annealing at 56°C and 1:40 minutes of elongation at 72°C. And at the end of 30 cycles: 15 seconds of denaturation at 90°C, 15 seconds of primer anneal at 56°C and 1:40 minutes of elongation at 72°C. A final elongation was performed for 10 minutes at 72°C.

### Viral load and CD4 testing

Plasma viral load (VL) was assessed by branched DNA (b-DNA) technology (Versant HIV-1 RNA 3.0; Bayer Co., Tarrytown, NY) with a detection limit of 50 HIV-1 RNA copies/ml. CD4+cells from peripheral blood were measured by cytometry (Coulter XL; Coulter Co., Hialeah, FL, USA).

### Resistance analysis

Sequences were analyzed to identify mutations associated with reduced susceptibility to protease and RT inhibitors, as reported by the International AIDS Society-USA in 2010 [36]: RT–M41L, A62V, K65R, D67N, 69 insert, K70R, L74V,V75I, F77L, L100I, K101P, K103N, V106A, V106M, V108I, Y115F, F116Y, Q151M, Y181C, Y181I, M184V, M184I, Y188C, Y188L, Y188H, G190A, G190S, L210W, T215Y, T215F, K219Q, K219E and P225H; protease–D30N, V32I, M46I, M46L, I47A, I47V, G48V, I50L, I50V, I54L, I54M, Q58E, L76V, V82A, V82F, V82L, V82S, V82T, N83D, I84V, N88S and L90M.

### Phylogenetic analysis

Sequence alignment was performed by CLUSTAL W (BioEdit 7.1.3.0 sequence alignment editor [37]). Neighbour-joining (NJ) trees were constructed under the Kimura 2-parameter model with the MEGA5 programme [38]. Sequences were individually analyzed by Simplot 3.5.1 [39] and recombination analysis was then performed by bootscanning analysis [39].

### Statistical analysis

Chi-square test and Fisher's exact test were used to compare proportions of resistance mutations and patient's epidemiological records.

## Results

A total of 197 newly HIV-1-diagnosed individuals were studied. The average age was 37 years. In 45 cases, the *pol* gene could not be successfully sequenced and were excluded from the analysis, resulting in a total of 152 analyzed samples (77%). Phylogenetic analyses showed predominance of two viral subtypes: 77 (50.6%) samples were recombinants between subtypes B and F, 70 (46.0%) were subtypes B and 5 (3.3%) were “non B-non BF” variants.

Twelve individuals (7.9%) were found to harbour primary resistance mutations, 12 males and 1 female. No significant associations were found between presence of variants with resistance mutations and patient's epidemiological records, CD4 count, VL or viral subtype (Supplementary Table 1). According to drug class, mutations associated with resistance to NNRTI were the most prevalent, being present in nine (5.9%) individuals. NRTI mutations and primary mutations associated with resistance to protease inhibitors (PIs) were both found each in two individuals (1.3%).

The most prevalent resistance mutations associated with NNRTI were K103N and G190A. Both were present in 88.9% (8/9) patients with primary resistance mutations for this class of drugs and accounts for the 66.7% (8/12) of the overall primary resistance and for 90% (9/10) of the NNRTI resistance. Regarding PI resistance mutations, they were found in two patients (Table 1) who shared the same mutations profile. One of them was a 32-year-old MSM man enrolled at the Site D, and the other was a 25-year-old bisexual man enrolled at the Site F. Phylogenetic analysis revealed a close relationship between both viral strains as evidenced by the monophyletic highly supported clade containing both sequences (bootstrap = 100, data not shown). While these two sequences are closely related, according to the phylogenetic analysis they are separated by a genetic distance of 0.062 showing that, in spite of sharing the same mutation profile, they are different from each other (Supplementary Figure 1).

**Table 1:** Epidemiological and virological characteristics of the 12 individuals harbouring resistance mutations

										Resistance mutations		
										
ID	Age group	Risk group	Site	First HIV-positive diagnosis	Sampling date	Clinical stage at diagnosis	Viral subtype	CD4 count (cells/µl)	Log_10_ (VL)	Protease inhibitor	Nucleoside RT inhibitor	Non-nucleoside RT inhibitor
1	50–55	HTS	A	05.27.2010	09.27.2010	Asymptomatic	BF	337	4.58	–	–	K103N
2	30–35	HTS	B	07.16.2010	09.16.2010	Symptomatic without AIDS criteria	B	132	5.87	–	–	G190A
3	35–40	MSM	A	12.01.2010	02.01.2011	Asymptomatic	B	218	4.66	–	A62V**, T215D*	–
4	35–40	HTS	C	11.01.2010	01.04.2011	AIDS	BF	146	2.85	–	–	G190A
5	30–35	MSM	D	08.20.2010	11.05.2010	Acute retroviral syndrome	BF	394	NA	M46I, L76V, V82A, I54V*	–	–
6	20–25	HTS	C	10.01.2010	10.13.2010	Asymptomatic	B	301	4.53	–	–	K103N
7	30–35	MSM	E	11.30.2010	02.08.2011	Asymptomatic	B	494	4.92	–	–	K101P**
8	30–35	HTS	D	02.10.2011	02.28.2011	AIDS	BF	74	NA	–	–	K103N
9	20–25	Bisexual	F	08.17.2010	09.23.2010	Asymptomatic	BF	308	NA	M46I, L76V, V82A, I54V*	–	–
10	35–40	MSM	E	12.14.2010	02.08.2011	NA	B	22	4.49	T69D*	D67N, K70R, M184V, T215F, K219Q	G190A, K101E*
11	40–45	NA	C	05.05.2010	11.02.2010	Symptomatic without AIDS criteria	B	473	4.85	–	–	K103N
12	45–50	MSM	E	12.14.2010	02.15.2011	Asymptomatic	BF	363	5.65	–	–	G190A

* Considered resistance mutations only by the WHO guidelines.

** Considered resistance mutations only by the IAS guidelines.

NA = not available, VL = viral load, RT= reverse transcriptase.

**Figure 1:**
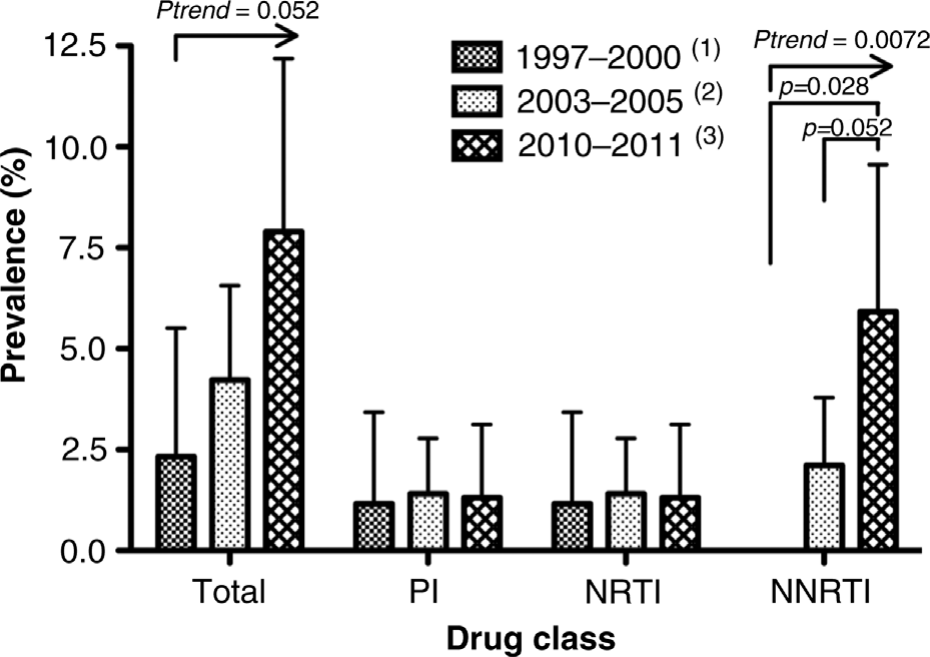
Prevalence of primary resistance in the present study compared with our previous studies and according to drug class. Arrows indicate significant trends in time according to Chi-square test for trend. (1) Kijak *et al*. [31]. (2) Dilernia *et al*. [32]. (3) Current study.

When we compared these results with our previous studies, as shown in Figure 1, we observed an almost significant increase in NNRTI resistance compared with data collected in 2003–2005 (*p*=0.052) and a significant increase compared with data collected in 1997–2000 (*p*=0.028). The overall trend across the 10-year period was significant for NNRTI resistance (Chi-square for trend, *p*=0.0072) and almost significant for overall resistance (Chi-square for trend, *p*=0.052).

Finally, we re-analyzed our dataset using the list of mutations reported by the WHO guidelines [21]. Among patients with no evidence of primary resistance based on the IAS list, none of them presented resistance mutations based on the WHO list. Among the 12 patients with evidence of primary resistance based on the IAS list, 11 of them presented resistance mutations according to the WHO guidelines while patient ID#7 (Table 1) exhibited the mutations K101P that is not considered by the WHO guidelines.

## Discussion

Based on our results, 7.9% (CI: 3.6–12.2, *n*=152) of individuals newly diagnosed between 2010 and 2011 in Buenos Aires harbour major resistance mutations associated with reduced susceptibility to ARV drugs. This figure is similar to that observed in other developing countries [7–14] but still lower than the prevalence observed in many developed settings [15–19, 21].

Previously, we had reported a prevalence of 2.3% (CI: 0–5.5, *n*=86) primary resistance in a similar population diagnosed between 1997 and 2000 [32], and 4.2% (CI: 1.9–6.5, *n*=284) [32] for 2003–2005. Comparing prevalence in the first study with the one reported in the present study, we observed an increase of almost four-folds (*p*=0.028) in 10 years. When data were analyzed separately according to drug class, we observed that the mentioned trend was driven by the increase in NNRTI resistance mutations for which the prevalence resulted increased from no detection of those mutations in the first period to a prevalence of 2.1% in 2003–2005 and 5.9% in 2010–2011 (Chi-square for trend, *p*=0.0072). At the mutation level, K103N represents around the 50% of NNRTI primary resistance in the last two periods while the remaining 50% was dominated by the V108I mutation in the previous study and by the G190A mutation in the present study. It is important to highlight that, as reported by June 2010, in Buenos Aires the 86.6% of patients treated with NNRTIs were under efavirenz-based regimens and the remaining 13.3% were under nevirapine-based regimens [40], which are significantly impaired by K03N mutation. Therefore, it is likely that ARV treatment constitute indeed the population-level selective force driving persistence of this mutation in our population since K103N is not a natural polymorphism of the virus circulating in our region. Interestingly, we found a high prevalence of G190A, not previously reported in the region; and the absence of V108I, found about equally often as K103N in our previous study during 2003–2005.

It is important to highlight that in the present study we did not considered any upper limit for the age at enrolment while the 2008 WHO HIVDR guidelines, as well as the 2012 update, recommend restricting the inclusion criteria to patients younger than 25 years in order to minimize the proportion of chronically HIV-positive individuals and to obtain a better estimate of transmitted resistance. Applying that restriction to our study results in a similar estimation or resistance (7.1%) (Supplementary Table 1), in contrast to previous studies that have found significantly different prevalence in that age group [41]. However, the limited sample size of patients younger than 25 limits any further analysis.

According to our results, during the course of the past 10 years a significant increase in primary resistance to NNRTIs has occurred. Although prevalence remains lower than in developed settings, our study shows that accumulation of ARV resistance mutations can be a significant public health concern even in settings where the proportion of individuals living with HIV under treatment is limited.
